# Effect of conventional cigarette smoking and recent heated tobacco products on CAD/CAM restorative materials

**DOI:** 10.1186/s12903-024-04423-2

**Published:** 2024-07-05

**Authors:** Fatma Makkeyah, Omar El Sergany, Mohamed Shamel, Mahmoud Al Ankily

**Affiliations:** Faculty of Dentistry, The British University in Egypt, Cairo, Egypt

**Keywords:** Cigarette smoking, Heated tobacco products, Surface roughness, Color stability

## Abstract

**Objective:**

To determine the effects of conventional cigarette smoking (CS) and recent heated tobacco products (HTPs) on the surface roughness and color stability of different indirect restorative materials.

**Materials and methods:**

One hundred disc-shaped samples were constructed of three different restorative CAD/CAM materials: lithium disilicate glass–ceramic (IPS e.max CAD; Ivoclar Vivadent, Liechtenstein), zirconia (BruxZir® Zirconia, Glidewell, USA) and polyetheretherketone (BioHPP® bredent GmbH, Germany). Of the IPS e.max CAD and the Bruxzir samples, 20 samples were glazed, and 20 samples were polished, while the BioHPP samples were all polished according to the manufacturer’s instructions.

Fifty samples were subjected to conventional cigarette smoking (LM, Philip Morris International Inc., Egypt) (Groups: IPS e.max CAD_Glazed exposed to CS (LD_G_Cig), IPS e.max CAD_Polished exposed to CS (LD_P_Cig), Bruxzir_Glazed exposed to CS (Zr_G_Cig), Bruxzir _Polished exposed to CS (Zr_P_Cig) and BioHPP exposed to CS (PEEK_Cig) and fifty samples were exposed to heated tobacco product smoking (Heets, Russet selection, Philip Morris International Inc., Italy) (Groups: IPS e.max CAD_Glazed exposed to HTP (LD_G_HTP), IPS e.max CAD_Polished exposed to HTP (LD_P_HTP), Bruxzir_Glazed exposed to HTP (Zr_G_HTP), Bruxzir CAD_Polished exposed to HTP (Zr_P_HTP) and BioHPP exposed to HTP (PEEK_HTP).. Six hundred cigarettes/heets representing 30 days of medium smoking behavior (20 cigarettes/day) were used. Before and after exposure to smoke, the surface roughness of all the samples was measured using JITAI8101 surface roughness tester (Beijing Jitai Tech Detection Device Co., Ltd, China, and the color parameters were assessed using VITA Easyshade Advance 4.01 (VITA shade, VITA made, VITA). The data were analyzed using One-way ANOVA, paired sample t-test and independent sample t-test. The significance level was set at α < 0.05.

The surface topography was evaluated by scanning electron microscopy (SEM) and analyzed using energy-dispersive X-ray (EDX) spectroscopy to determine changes in the surface chemical composition.

**Results:**

Both types of smoking caused significant increases in the surface roughness of all the samples. There was a significant difference in color change between CS and HTP for all materials with different surface finish (*P* < 0.01) and zirconia had the greatest effect on color change (*P* < 0.001). In contrast, polyetheretherketone (PEEK) “BioHPP” had the least effect (*P* < 0.001).

**Conclusion:**

Exposure to different types of smoking induce changes in the surface topography and color of different esthetic restorative materials. Compared with HTP, conventional cigarette smoke has a greater effect on the surface roughness and color stability of esthetic restorative materials. The glazed surfaces showed less change in surface topography than did the polished surfaces. Zirconia showed better color stability when compared to polyetheretherketone (PEEK).

## Background

Due to their excellent esthetics, wear resistance, and biocompatibility, dental ceramics are widely used in prosthetic dentistry. Ceramics can replace tooth structures that are missing or damaged, such as fixed partial dentures, complete and partial dentures, and other structural components [1–3]. The use of computer-aided design/computer-aided machining (CAD/CAM) systems in the fabrication of dental restorations provides increased precision, increased efficiency and enhanced strength [4]. A wide variety of materials have been developed for milling that ultimately contribute to the longevity of the restoration. Among these materials are zirconia-based and glass-based ceramics [5, 6].

Zirconia-based ceramics are widely sought upon for clinical use because of their remarkable physical properties, such as high wear and corrosion [7]. They can be used in different prosthetic procedures, such as crowns, fixed partial dentures, dental implants, and abutments [7]. Lithium disilicate glass–ceramic is another widely used material. It provides highly esthetic restorations owing to its crystalline components, which enhance light transmission and mechanical properties [8]. The block is partially crystallized to facilitate machining, and the desired mechanical strength and optical characteristics, such as tooth color and translucency, are fully revealed after crystallization [9]. Another powerful biomaterial that has been increasingly used in dentistry is polyetheretherketone (PEEK), which is a tooth-colored material with a high-performance semicrystalline structure that has good physical properties, such as high resilience and strength. Additionally, PEEK is lightweight and can survive longer in harsh environments. It has trauma or shock-cancelling abilities, fracture-resisting abilities, stress-distributing abilities and osseointegrating abilities [10]. These properties have led to an increased demand for materials on the market that can be used for removable and fixed partial dentures and dental implants [10].

Nearly 1.3 billion individuals worldwide has been reported to smoke [11], which is of great public concern. The inhalation of toxic chemicals/components, such as tar, nicotine and carbon monoxide, that could contribute to preventable illnesses and premature death [12, 13]. Because cigarette smoke contains a high concentration of harmful components that must dissolve in oral fluids before entering the systemic circulation, it is a major cause of several dental problems, including periodontal diseases [14], dental implant failure [15], tooth or restoration pigmentation [16–18], precancerous lesions [19], oral cancer, dental caries [20] and alteration of taste [21]. Furthermore, it has been demonstrated that cigarette smoke changes physical characteristics and increases the surface roughness of dental restorations [16].

Although cigarettes remain the most widely used form of tobacco, new products such as heated tobacco products (HTPs) are gaining popularity [22]. Therefore, in the era of novel tobacco and nicotine products, it is pertinent to identify which components of tobacco cause staining and what the true staining potential of the novel products is. Smoke that emerges directly from a lighted cigarette is frequently referred to as "whole smoke." It is made up of liquid droplets suspended in an aerosol combination of gases and semivolatile chemicals [23]. This particulate phase is commonly called the particulate phase. It is commonly known as "tar" or nicotine-free particulate fraction when it is devoid of nicotine [23]. This tar builds up on cigarette filters, turning them yellow‒brown, suggesting that these tar components stain the dental structures and restorations [24]. E-cigarettes emit aerosols that include nicotine and other substances, but they do not produce the same particulate matter as traditional cigarettes. HTPs are based on the principle that burning tobacco is unnecessary to liberate nicotine. Processed tobacco is heated without reaching ignition to produce an emission containing nicotine and other chemicals, which is then inhaled by users [25]. The release of nicotine is attained in HTPs by volatilization and even pyrolysis [26]. The mainstream emission from HTPs seems to deliver less nicotine per stick than a conventional cigarette. In studies, nicotine in mainstream emission ranged from 57–83% of that of a reference cigarette [27]. Furthermore, it has been demonstrated that HTPs produce less particulate matter than cigarette smoke does [27].

A line of argument is that these products, as a result, may cause less staining when compared to conventional smoking. Manufacturers have often promoted these novel products with claims including, “no smelly clothes” or “no yellow teeth” [28]. These are cosmetic rather than health‐based claims and are subject to less stringent regulations. In 2020, Vohra et al., evaluated the effect of electronic nicotine delivery systems (ENDS) aerosol when compared to conventional cigarette smoke (CS) on the color stability of dental ceramic (DC) and resin composite (RC), they found that both types of smoking caused similar discoloration levels [29]. Few studies [29, 30] assessed the effect of cigarette smoke on ceramics. The effect of CS compared with a non-exposure control on dental ceramics was explored and the results showed evidence that CS caused slightly more staining on ceramic in comparison to the control. Also, few studies [24, 31–34] assessed the effects of HTPs in comparison with CS on dental staining and evidence was found that HTPs caused less dental staining when compared with CS. However, there is a lack of information regarding the effect of HTPs in comparison with CS on commonly used dental ceramics.

The significant increase in the use of these novel tobacco and nicotine products especially in young individuals, makes it essential to identify the effect of these novel tobacco and nicotine products on the commonly used dental restorations.

Therefore, this study aims to determine the effect of conventional cigarette smoking (CS) and recent heated tobacco products (HTP) on the surface roughness and color stability of different indirect restorative materials.

## Materials and methods

### Samples preparation

This research project protocol was reviewed and approved by the Research and Ethics Committee of the Faculty of Dentistry, The British University in Egypt, with project no. 23–058.

In this study, the samples were exposed to two different types of smoking: conventional cigarette smoking (LM, Philip Morris International Inc., Egypt) and heated tobacco products and HTP (Heets, Russet selection, Philip Morris International Inc., Italy). One hundred disc-shaped samples 10 mm in diameter and 2 mm in thickness were constructed according to the manufacturer’s instructions. Three different restorative CAD/CAM materials were used: lithium disilicate glass–ceramic (IPS e.max CAD; Ivoclar Vivadent, Liechtenstein) (60 samples), zirconia (BruxZir® Zirconia, Glidewell, USA) (60 samples) and polyetheretherketone (BioHPP® bredent GmbH, Germany) (30 samples). Of the IPS e.max CAD and the Bruxzir samples, 20 samples were glazed, and 20 samples were polished, while the BioHPP samples were all polished according to the manufacturer’s instructions.

The samples were then assigned to 10 groups: 1: IPS e.max CAD_Glazed exposed to CS (LD_G_Cig), 2: IPS e.max CAD_Glazed exposed to HTP (LD_G_HTP), 3: IPS e.max CAD_Polished exposed to CS (LD_P_Cig), 4: IPS e.max CAD_Polished exposed to HTP (LD_P_HTP), 5: Bruxzir_Glazed exposed to CS (Zr_G_Cig), 6: Bruxzir_Glazed exposed to HTP (Zr_G_HTP), 7: Bruxzir _Polished exposed to CS (Zr_P_Cig), 8: Bruxzir CAD_Polished exposed to HTP (Zr_P_HTP), 9: BioHPP exposed to CS (PEEK_Cig) and 10: BioHPP exposed to HTP (PEEK_HTP).

### Measurements and assessments

The surface roughness and color parameters of all the samples were assessed before and after the smoking exposure experiment. Surface roughness was measured using profilometer (JITAI8101 Surface Roughness Tester—Beijing Jitai Tech Detection Device Co., Ltd., China). Each sample was measured three times at different areas (in the middle and sides), and the average was calculated to determine the mean values of the surface roughness (Ra) following ISO 11562 recommendations for standardization [35–37]. Color parameters were measured using a VITA Easyshade Advance 4.01 digital spectrophotometer (VITA shade, VITA made, VITA). The three-dimensional (3D) parameters of the color were recorded numerically: L*, a*, and b* values, where L is the axis of lightness, a is the value representing the axes of chromaticity (green‒red), and b is the value representing the axes of color (blue‒yellow). The color change (∆E) was calculated according to the following formula: ΔE_2-1_ = ([ΔL]^2^ + [Δa]^2^ + [Δb]^2^)^1/2^ [38].

### Scanning electron microscopy (SEM–EDX)

One sample from each group was examined by scanning electron microscopy (Thermo Fisher (USA) Quattro S Felid Emission Gun, Environmental SEM “FEG ESEM”) at the Nanotechnology Research Center at The British University in Egypt to evaluate the surface topography. It was analyzed using energy-dispersive X-ray (EDX) at two different points to determine changes in surface chemical composition.

### Smoking exposure experiment

A smoking standardizing device was specially designed to simulate the smoking process. It consisted of a gearbox to reduce the speed of the motor to 2 Hz (2 cycles per second) with a crankshaft and connecting rod attached to a slider to change the rotation movement into linear movement (A) of 4.5 cm length. A 12 cm (6 cm radius) stainless steel cylinder with a piston (B) was used to create suction power and a volume of 500 ml, which simulated the tidal volume during smoking. A cigarette or electronic smoking device was attached to a valve that permits inhalation of smoke in one direction, simulating the mouth, and another valve simulating the nose, allowing exhalation to occur exclusively in one direction (C). A pool of water with a heater (D) connected to a thermal sensor regulates the temperature from 36.5 to 37.5 °C and 100% humidity, simulating the oral cavity (E). The samples (B) were mounted on two perforated trays to allow full exposure of all the samples to smoke (F).

**Fig. 1:**
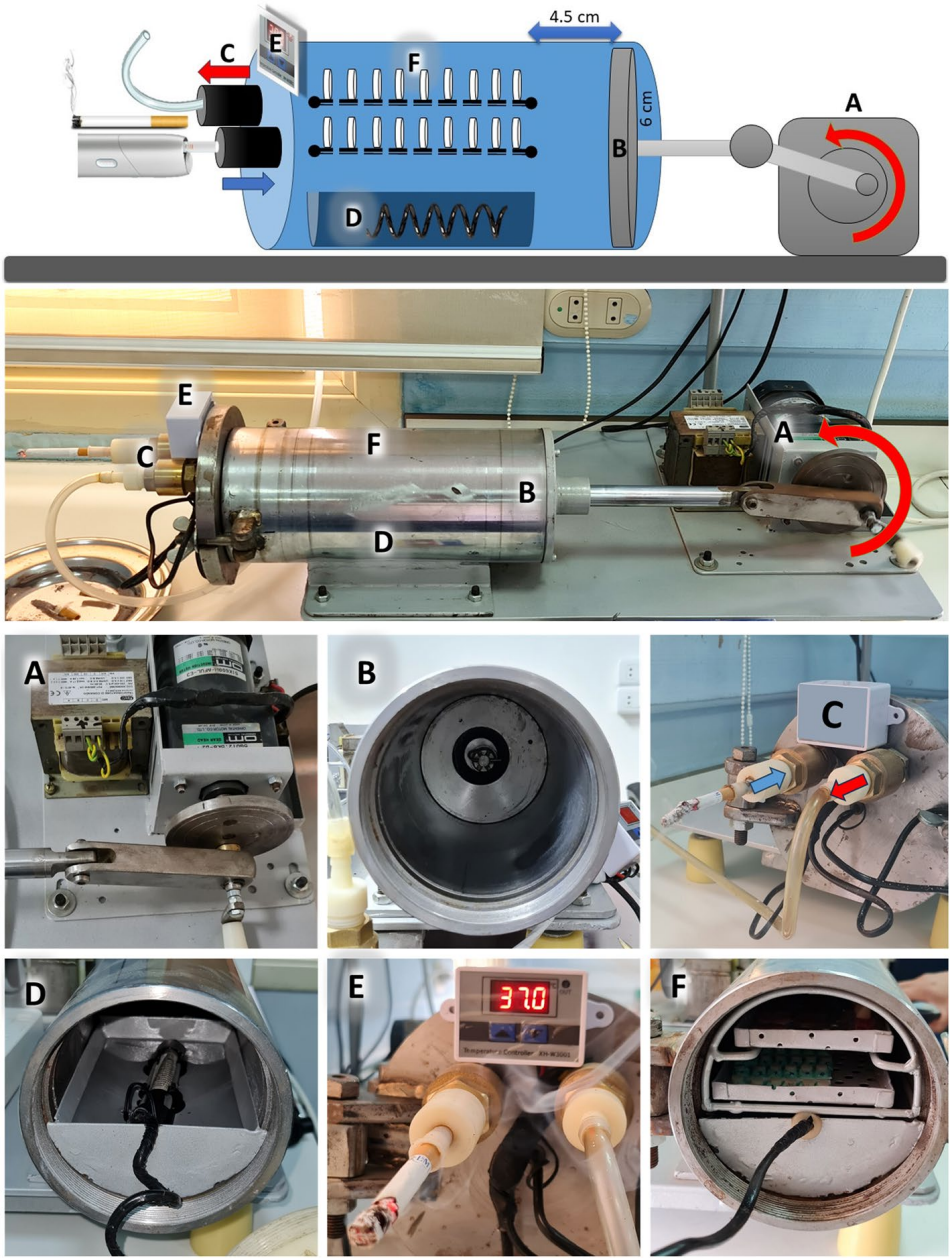
Smoking standardizing apparatus with a motor and gearbox with a crankshaft (**A**), a piston (**B**), two valves that allow inhalation and exhalation in one direction only (**C**), a pool of water with a heater (**D**) connected to a thermal sensor (**E**), and two perforated trays (**F**)

The samples were exposed to 600 cigarettes/heets, representing 30 days of medium smoking behavior (20 cigarettes/day). Then, the samples were gently washed with distilled water for 1 min Fig. 1.

### Statistical analysis

Statistical analysis of the obtained data was performed using SPSS for Windows (version 26.0; SPSS Inc., Chicago, IL, USA). Paired sample t-test was performed to determine the change in surface roughness. Independent sample t-test was used to compare the effects of different types of smoking and different surface finishing methods on the surface roughness and color change of each material. One-way ANOVA was used to determine the effect of different materials. Multiple linear regression was performed between the material, smoking type and surface finish to determine the most effective factor in the change in surface roughness and color. Significance level at α < 0.05.

## Results

**Fig. 2:**
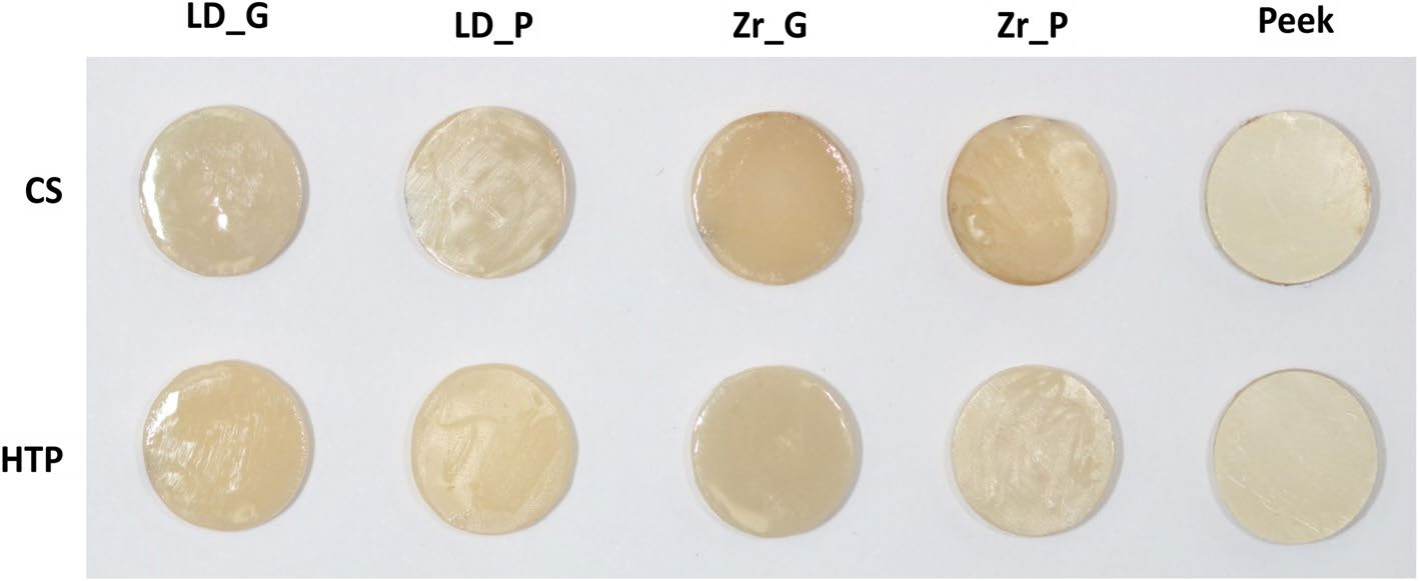
Samples after exposure to CS and HTP

Generally, the CS samples had a more pronounced color change than the HTP samples when observed by the naked eye (Fig. 2).

For the change in surface roughness, paired sample t-test showed a significant increase in the surface roughness of all groups before and after exposure to smoke (Table 1).

**Table 1:** Paired sample t-test for surface roughness before and after the exposure of samples

Groups	Paired differences					
	**Mean**	**Std. deviation**	**95% Confidence interval of the difference**		**t**	**Sig. (2-tailed)**
			**Lower**	**Upper**		
**LD_Glazed_CS**	0.19960	0.24036	0.02765	0.37155	2.626	0.028
**LD_Polished_CS**	0.89397	0.59332	0.46953	1.31840	4.765	0.001
**LD_Glazed_HTP**	0.08350	0.10258	0.01012	0.15688	2.574	0.030
**LD_Polished_HTP**	0.23377	0.19359	0.09528	0.37225	3.819	0.004
**Zr_Glazed_CS**	0.24387	0.16975	0.12243	0.36530	4.543	0.001
**Zr_Polished_CS**	0.74240	0.71085	0.23389	1.25091	3.303	0.009
**Zr _Glazed_HTP**	0.19627	0.19125	0.05945	0.33308	3.245	0.010
**Zr _Polished_HTP**	0.14997	0.10422	0.07541	0.22452	4.550	0.001
**PEEK_CS**	0.14307	0.16761	0.02316	0.26297	2.699	0.024
**PEEK_HTP**	0.09247	0.10532	0.01713	0.16781	2.776	0.022

**Fig. 3:**
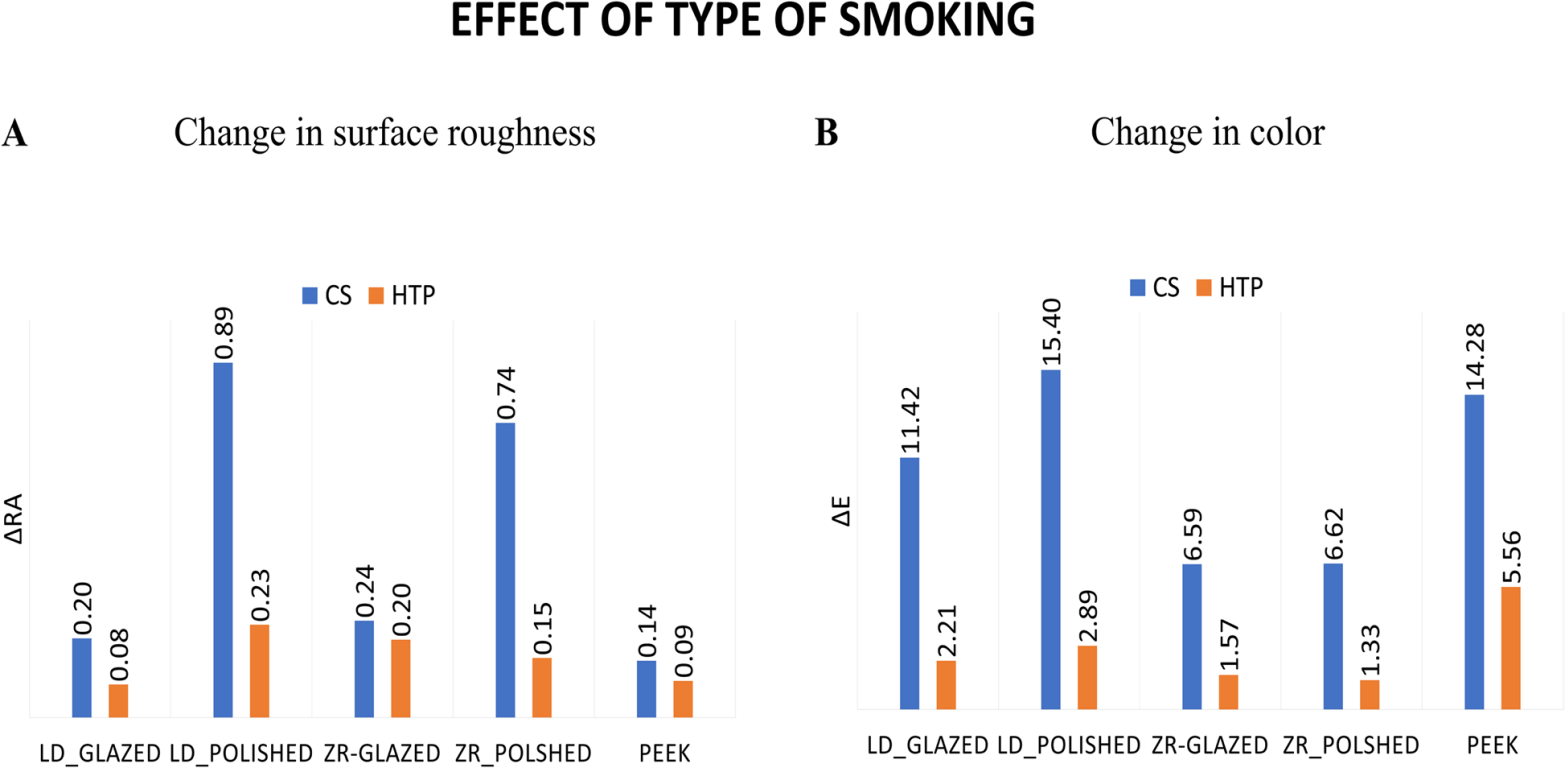
Effect of type of smoking on the change in surface roughness (**A**) and change in color (**B**)

When comparing the effects of different types of smoking, HTP smoking had a significantly lower roughness than cigarette smoking in the LD_Polished (*P* = 0.007) and Zr_Polished (*P* = 0.027) groups. None of the other groups showed a significant difference between cigarette and HTP smoking (Fig. 3A). However, for the color change, there was a significant difference in color change between CS and HTP for all materials with different surface finish (*P* < 0.01) (Fig. 3B).

**Fig. 4:**
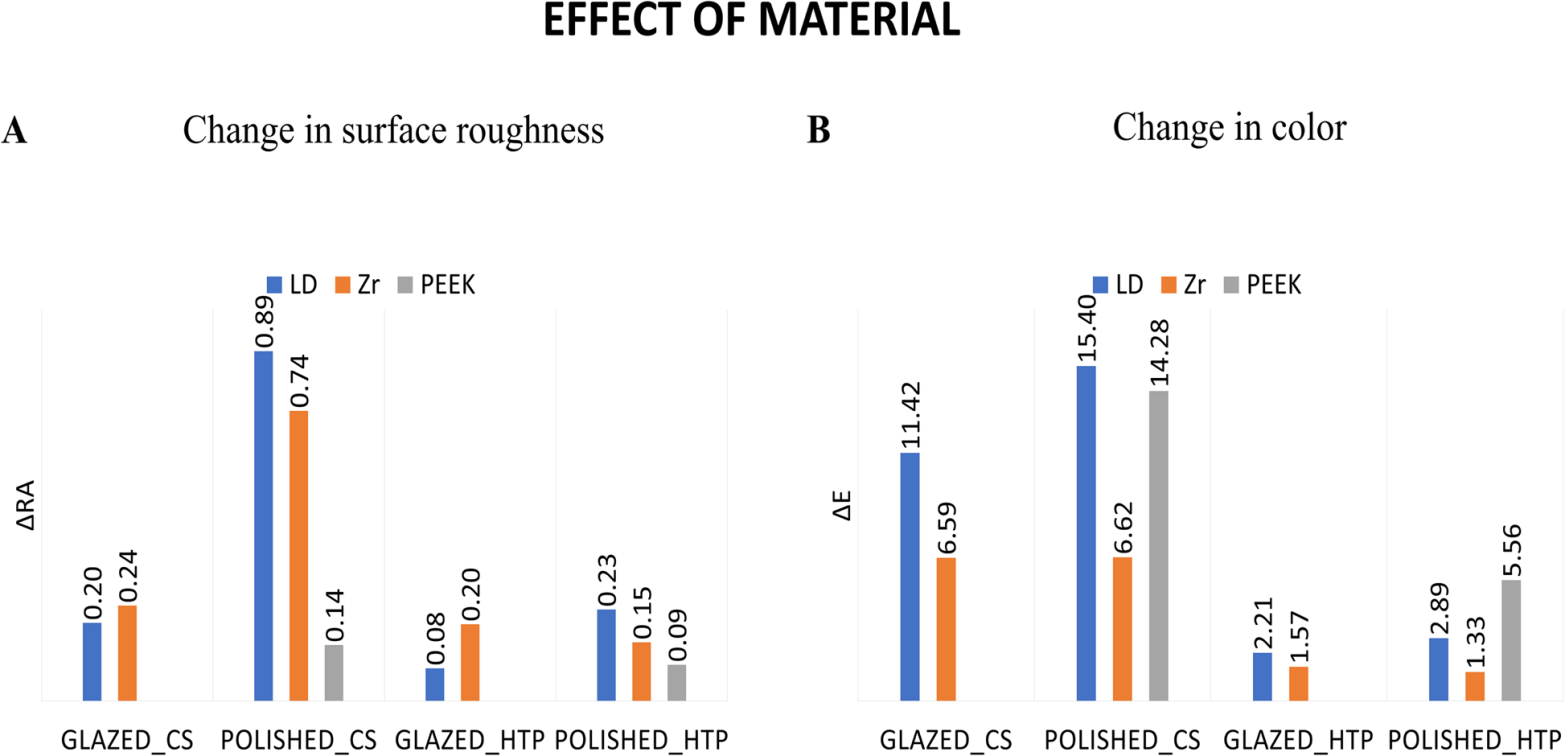
Effect of material on the change in surface roughness (**A**) and change in color (**B**)

Concerning the effect of the material, with CS, the PEEK group showed the least change in surface roughness compared to the LD_P and Zr_P groups, while the LD and Zr groups showed no significant differences. For HTP, there was no significant difference between the different materials (Fig. 4A). The zirconia groups with both types of surface finish showed significantly lower color changes than both the IPS e.max CAD and PEEK groups within the cigarette smoking groups (*P* < 0.001), while within the HTP smoking groups, both zirconia and IPS e.max showed significantly lower color changes from the PEEK group (*P* < 0.001) (Fig. 4B).

**Fig. 5:**
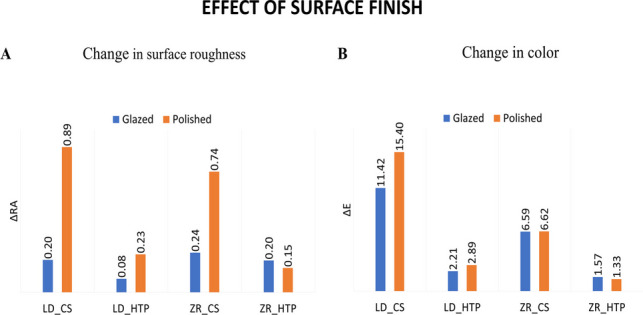
Effect of surface finish on the change in surface roughness (**A**) and change in color (**B**)

For the effect of surface finish, the surface roughness of the LD_G group was significantly greater than that of the LD_P group in the CS group (*P* = 0.005). In contrast, the difference between the two types of surface finish was not significant in the HTP groups (*P* = 0.05). However, the zirconia samples showed no significant change (Fig. 5A). There was no significant difference in color change between glazed and polished surfaces for any of the materials with different types of smoking (Fig. 5B).

Multiple linear regression analysis using stepwise technique showed that the change in surface roughness was affected by the type of smoking and surface finish, while the material was not influential (*r*^2^ = 0.19, df = 2, *F* = 13.5, *P* < 0.001); HTP caused a significantly smaller change in roughness than cigarette smoking (ß = -0.253), and the polished surface caused a significantly greater change in roughness (ß = 0.216). The color change of the samples was affected both by the type of smoking and the material, while the surface finish was not influential (*r*^2^ = 0.67, df = 3, *F* = 64.941, *P* < 0.001). HTP caused a significantly smaller change in color (ß = -8.148) than cigarette smoking, zirconia caused a significantly smaller change in color than did PEEK (ß = -5.889), and lithium disilicate caused a significantly smaller change in color than did PEEK (ß = -1.936).

### SEM–EDX

**Fig. 6:**
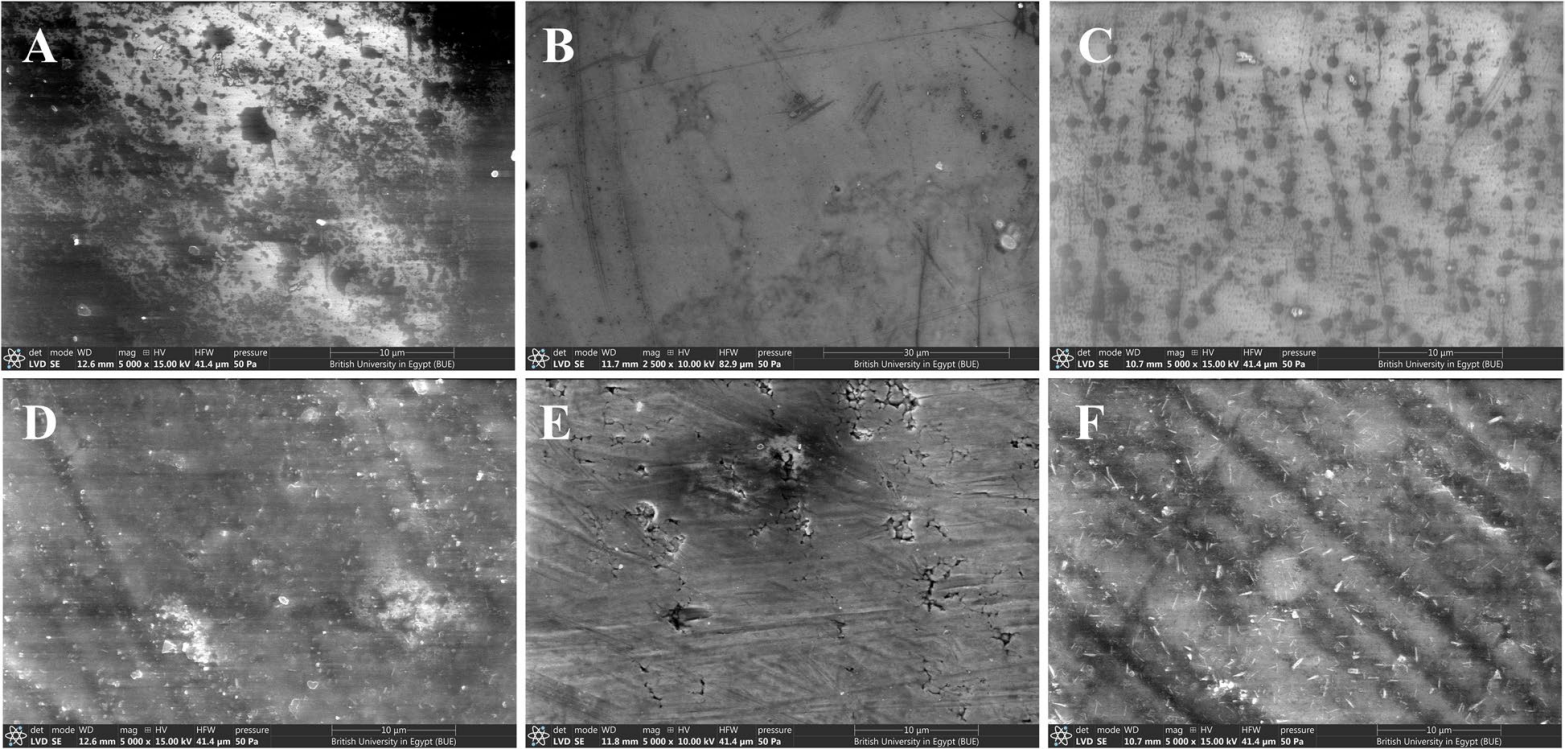
SEM of IPS e.max CAD samples; **A**: LD_Glazed_no exposure, **B**: LD_Glazed_CS, **C**: LD_Glazed_HTP, **D**: LD_Polishd_no exposure, **E**: LD_Polished_CS, **F**: LD_Polished_HTP

**Fig. 7:**
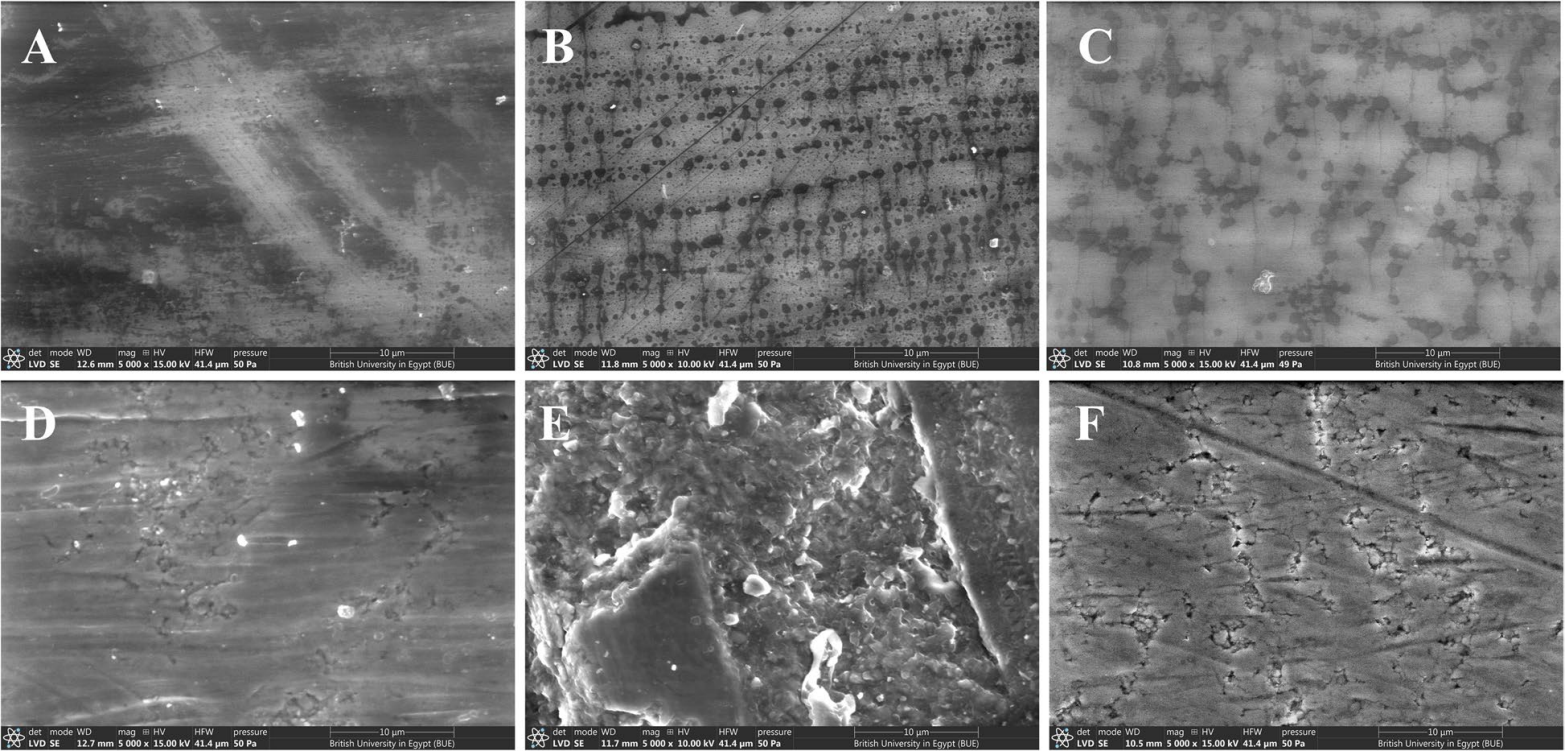
SEM images of the Bruxzir zirconia samples; **A**: Zr_Glazed_no exposure, **B**: Zr_Glazed_CS, **C**: Zr_Glazed_HTP, **D**: Zr_Polishd_no exposure, **E**: Zr_ Polished_CS, **F**: Zr_Polished_HTP

**Fig. 8:**
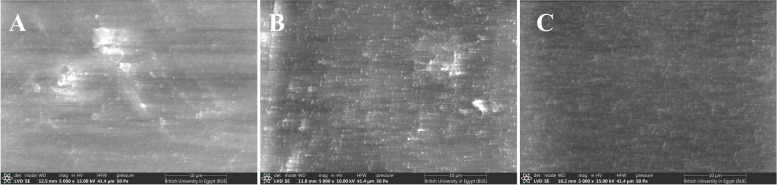
SEM of PEEK samples; **A**: PEEK_no exposure, **B**: PEEK_CS, **C**: PEEK_HTP

The surface topography images of the studied samples at 5000X are presented in Figs. 6, 7 and 8. The images of the glazed surfaces of the IPS_e.max_CAD and Bruxzir_Zirconia samples showed pitted surfaces, which may be due to the deposition of organic materials on the surface. The images of the polished surface of both materials that were exposed to cigarette smoking showed the most irregular topography with deep areas. The images of the PEEK samples showed a relatively small roughness with white spots.

The EXD analysis did not show detectable changes in the surface components of either IPS e.max CAD or Bruxzir_Zirconia before and after exposure to different types of smoking (Table 2).

**Table 2:** Shows the weight % of different surface components of IPS e.max CAD and Bruxzir_Zirconia before and after different types of smoking

		Before exposure	After cigarette smoking	After HTP smoking
Ex_Glazed	Oxides	54.515	58.075	54.68
	Silica	45.22	41.44	44.985
	Zirconia	0.265	0.485	0.335
Ex_Polished	Oxides	51.38	53.82	51.11
	Silica	43.475	41.27	43.51
	Zirconia	5.145	4.915	5.38
Zr_Glazed	Oxides	98.455	59.045	55.565
	Silica	45.32	40.645	44.105
	Zirconia	1.545	0.31	0.33
Zr_Polished	Oxides	30.075	36.405	28.26
	Silica	2.465	1.675	1.75
	Zirconia	69.925	61.915	69.99

## Discussion

Dental discoloration is the alteration of the natural tooth color. It can be classified based on the location of the discoloration or staining, into internal discoloration or external staining [39]. Tobacco smoking has long been considered a cause of external staining that can affect natural dentition as well as various dental restorations. Reducing the yellowing of teeth and restorations, observed in smokers, has often been used as a motivation to quit. The manufacturers of recent HTP claim reduced dental staining of these products. Hence, it has to be clarified how do tobacco and nicotine products affect the stainability of dental materials and hard tissues, the different staining levels of different tobacco and nicotine products and if any specific tobacco compounds have the potential to discolor teeth. So the objective of this study was to compare the effect of conventional CS and new HTP on surface roughness and color stability of three different esthetic restorative materials in their polished and glazed state. To simulate real-life conditions, a smoking device was constructed, and smoking was performed to simulate medium smokers' habits of 20 cigarettes per day for 30 days.

Conventional cigarettes are composed of tobacco, water, sugars, glycerol, propylene glycol, licorice extract, ammonium hydroxide, diammonium phosphate, cocoa, and natural and artificial flavors [40]. Nicotine represents 0.3%–4.26% of the weight of dried tobacco [41]. Cigarette smoke is made up of both a gaseous and a particulate phase, it contains toxic agents such as carbon monoxide (CO), carbon dioxide (CO_2_), air, water, and nicotine-free dry particulate matter (tar) that represents > 90% of smoke products [40, 42]. More than 99% of cigarette nicotine, which has a yellow shade, is contained in the particulate of CS [23]. It was suggested that color alteration of dental tissues and restorations in smokers is caused by the pigments contained in tobacco residue (tar) [42–44], and that the contact with CS gives a color mismatch between the dental surface and the restoration material [24]. Additionally, the smoking habit has deleterious thermal effects that have to be considered [45, 46].

The results of this study revealed that the surface topography of the restorations was affected negatively by both types of smoking and that the amount of change is related to the type of smoking and the surface finish of the material. Cigarette smoking showed significantly more ∆Ra than HTP for the polished surface of both zirconia and IPS e.max CAD. Cigarette smoking results in the combustion of elements resulting in the release of metals like arsenic, lead and cadmium along with dark components of smoke. These elements deposit on the surface, resulting in surface alterations and discoloration [47]. This finding may suggest that the glaze layer reduces the adherence of the byproducts produced by different types of smoking to the ceramic surface, while the polished surface retains more byproducts, especially with cigarette smoking leading to surface alterations. These surface alterations result in changes to the way light is reflected and transmitted through the material which affects the light scattering and how color is perceived visually [48, 49]. Our findings are in line with previous studies that exposure to smoke and tobacco products can trigger surface changes in ceramic restorations [50] and dental composites [16], leading to a reduction in the luminosity and a darker color in the specimens, with increased L* values and shift into redness and darker color clinically.

In this study, color change was found to be related to the type of smoking and material. Zirconia showed the least significant color change and the PEEK showed the most significant color change. This result was consistent with the results of Abhay et al., (2021) [51], who reported more color stability for zirconia than PEEK. The color change of IPS e.max CAD showed similar results to zirconia with HTP and similar results to PEEK with cigarette smoking suggesting an interaction between the material and type of smoking. The significantly more color change in the PEEK material is due to the microstructure of the materials which is a partially crystalline, thermoplastic high-performance polymer with a small grain size of the ceramic filler [52].

For all groups, cigarette smoking produced a more significant color change than HTPs smoking. These findings were consistent with the findings of Belli et al., (1997) [53] who reported evidence that cigarette smoke caused discoloration of dental ceramics. Additionally, according to the review by Paolone [54], all modified risk tobacco products (MRTP) presented significantly less color change than CS. Investigating the effect of HTPs and cigarette smoking, a systematic review and meta‐analysis by Karanjkar [55] found evidence that exposure to HTPs caused more staining of natural enamel and dentin when compared to a non-exposure [31–33], and that exposure to cigarette smoking caused slightly more staining on dental ceramics when compared to a non-exposure control [29, 30]. Also, they found that HTPs caused less dental staining when compared with cigarette smoke [24, 31–34].

Additionally, Haiduc [33] evaluated the pigments present in the total particulate matter (TPM) that was deposited on enamel by HTP aerosol and cigarette smoke. Eight terpenoids were identified using gas chromatography coupled to time-of-flight mass spectrometry following carbon disulfide extraction. These chemicals were identified in TPM extracts from cigarette smoke and heated tobacco product aerosol. TPM produced from CS stained tooth enamel more than TPM from aerosol. However, when samples exposed to nicotine-containing products were compared to control samples, nicotine was not found to be a significant cause of discolouration. At temperatures higher than 60 °C, nicotine dissolves readily in alcohol, ether, light petroleum, and water. When nicotine is in its base form, it combines with acids to generate salts that are usually solid and soluble in water, which can be cleaned with a toothbrush. They found that tooth discolouration is not significantly impacted by the nicotine in tobacco smoking. Additionally, a study on the whitening effects of chewing gum showed that medicated gums containing nicotine were effective in eliminating stains from teeth [56].

The color changes in esthetic restorations of ∆E < 1 cannot be detected by the naked human eye, 1 < ∆E < 3.3 can only be detected by a skilled dentist, and ∆E > 3.3 can be seen by anyone and is clinically unacceptable [46, 57]. The color change of zirconia and LD materials used in this study were found to be within the acceptable range when exposed to HTP while all other groups were found to be unacceptable.

A limitation of the present study was the lack of examination of different brands, nicotine concentrations, and brushing simulation during sample exposure to smoke to further evaluate the impact on dental restorative materials. Future studies are recommended on more clinically available restorative materials using different nicotine brands and concentrations.

## Conclusions

Within the limitations of this study, it was concluded that exposure to different types of smoking leads to degradation in the surface topography and esthetic appearance of different dental restorations. These effects were more pronounced with conventional cigarette smoking. Future studies are recommended on more clinically available restorative materials using different nicotine brands and concentrations.

## Data Availability

The datasets used and/or analysed during the current study available from the corresponding author on reasonable request.
